# Egg Consumption and Risk of Metabolic Syndrome in Korean Adults: Results from the Health Examinees Study

**DOI:** 10.3390/nu9070687

**Published:** 2017-07-02

**Authors:** Sangah Shin, Hwi-Won Lee, Claire E. Kim, Jiyeon Lim, Jong-koo Lee, Sang-Ah Lee, Daehee Kang

**Affiliations:** 1 Institute of Environmental Medicine, Seoul National University Medical Research Center, Seoul 03080, Korea; ssa8320@snu.ac.kr; 2 Department of Preventive Medicine, Seoul National University College of Medicine, Seoul 03080, Korea; hwiwon@snu.ac.kr (H.-W.L.); claireekim@snu.ac.kr (C.E.K.); jiyeonlim@snu.ac.kr (J.L.); 3 Department of Biomedical Sciences, Seoul National University Graduate School, Seoul 03080, Korea; 4 JW Lee Center for Global Medicine, Seoul National University College of Medicine, Seoul 03087, Korea; docmohw@snu.ac.kr; 5 Department of Family Medicine, Seoul National University Hospital, Seoul 03080, Korea; 6 Department of Preventive Medicine, Kangwon National University, Kangwon-do 24341, Korea; sangahlee@kangwon.ac.kr

**Keywords:** metabolic syndrome, egg consumption, the Health Examinees (HEXA) study, Korean

## Abstract

Metabolic syndrome (MetS) is defined as a cluster of metabolic alterations such as abdominal obesity, dyslipidemias, elevated fasting glucose, and hypertension. Studies on the association between egg consumption and MetS are limited and inconsistent. A cross-sectional analysis was conducted to examine the association of egg consumption with MetS among Korean adults aged 40–69 years. A total of 130,420 subjects (43,682 men and 86,738 women) from the Health Examinees Study were selected for the final analysis. Egg consumption was estimated using a validated 106-item food frequency questionnaire. MetS was defined using the National Cholesterol Education Program, Adult Treatment Panel III. Logistic regression analyses were performed to identify the association of egg consumption with MetS via odds ratios (ORs) and 95% confidence intervals (CIs) after adjusting for potential variables. Among 130,420 subjects, 34,039 (26.1%) people had MetS. Consumption of more than 7 eggs/week was associated with a lower odds of MetS risk compared to those who consumed less than one egg/week in women (OR: 0.77, 95%CI: 0.70–0.84, *p* trend < 0.0001). Higher egg consumption was inversely associated with the MetS components: elevated waist circumference (OR: 0.80, 0.75–0.86), elevated triglyceride (OR: 0.78, 0.72–0.85), reduced high-density lipoprotein cholesterol (HDL-C) (OR: 0.82, 0.77–0.88), elevated blood pressure (OR: 0.86, 0.80–0.92), and elevated fasting glucose (OR: 0.94, 0.83–0.99) in women; reduced HDL-C (OR: 0.89, 0.80–1.00) in men. Our results suggest that higher egg consumption may be associated with a reduction in the odds for MetS and all five metabolic components in women, and the risk of reduced HDL-C in men.

## 1. Introduction

Metabolic syndrome (MetS) is a clustering of at least three of the five following metabolic alterations: elevated waist circumference, high serum triglyceride, low high-density lipoprotein cholesterol (HDL-C), elevated fasting plasma glucose, and elevated blood pressure (BP) [1]. MetS is associated with the risk of developing cardiovascular disease (CVD) and type 2 diabetes [2]. Although the etiology of MetS is not well understood, various factors such as genetic, metabolic, and environmental risk may contribute to the development of MetS [3]. 

Among the dietary factors, eggs are most often recognized as a relatively rich source of dietary cholesterol. Findings from some observational studies suggest that higher egg consumption in adults was associated with an increased risk of diabetes in men and women [4,5]. On the other hand, eggs are a crucial source of many essential nutrients, such as protein, unsaturated fatty acids, minerals (calcium, phosphorus, potassium, and choline), and fat-soluble and B vitamins [6]. This has led to discrepancies in dietary recommendations across populations and inconsistent results for egg consumption and the risk of MetS or its components. Previous prospective studies in adults found decreased risk of diabetes [7] or no association for diabetes [8,9] or CVD [10]. One meta-analysis using fourteen studies in USA, other western countries, and China reported a dose-response positive association between egg consumption and the risk of CVD and diabetes [11]. By contrast, a recent meta-analysis using several prospective studies showed that higher egg intake was not related to the risk of CVD in subjects without type 2 diabetes, but associated with an increased risk of type 2 diabetes among non-diabetic patients and CVD comorbidity among diabetic patients [12]. In randomized controlled trials, an increase in egg consumption in diet resulted in reduced plasma insulin and insulin resistance and improved the atherogenic lipoprotein profile with MetS [13]. Another clinical study reported that a diet high in cholesterol obtained from eggs improved glycaemic and lipid profile, blood pressure, and apo-B in individuals with type 2 diabetes [14]. 

Until now, the association between egg consumption and cardiometabolic risk factors are still controversial and the results regarding the association of egg consumption with MetS have been restricted. Therefore, it is important to investigate the relationship between egg consumption and the risk of MetS and its components. Therefore, the current study aimed to identify the association of egg consumption with prevalent MetS and its components among Korean adults aged 40–69 in the Health Examinees (HEXA) study.

## 2. Subjects and Methods

### 2.1. Study Population

The HEXA study is a large-scale genomic community-based study conducted in Korea from 2004–2013. For baseline recruitment, eligible participants were asked to volunteer through on-site invitation, mailed letters, telephone calls, media campaigns, or community leader-mediated conferences. A total of 169,722 subjects aged between 40–69 years old were recruited in 38 general hospitals and health examination centers in eight regions in Korea. The HEXA study design has been described in detail in previous studies [15,16].

**Figure 1:**
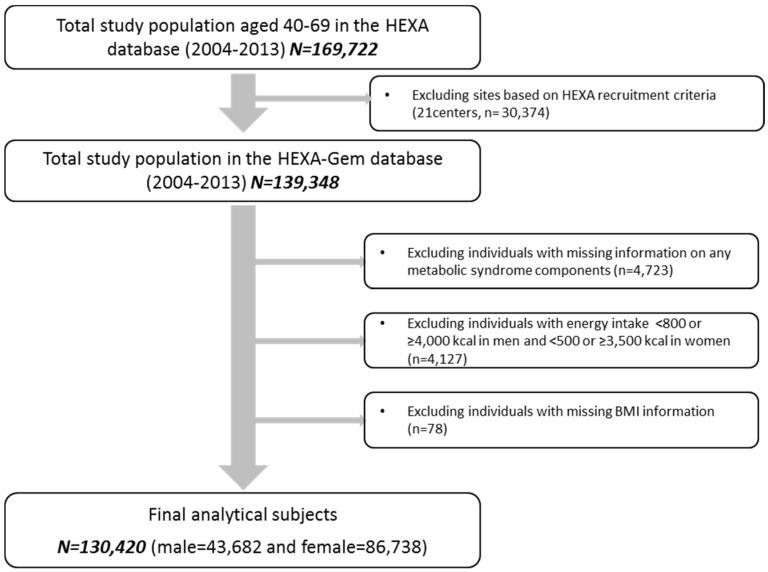
Flow diagram of analytical sample in the current study. HEXA, Health Examinees.

Updated from the previously published HEXA studies, the current study uses the HEXA-G (Health Examinees-Gem) participant sample which was given additional eligibility criteria on the participating sites (i.e., health examination centers and training hospitals). Of the original 38 sites, HEXA-G applied exclusion criteria of the following: (1) 8 sites (*n* = 9370) that only participated in the pilot study years 2004–2006; (2) 8 sites (*n* = 12,205) that did not meet the HEXA biospecimen quality control criteria (i.e., different testing protocols); (3) 5 sites (*n* = 8799) that have participated in the study for less than 2 years. In the new HEXA-G data, a total of 139,348 participants remained. Among the HEXA-G subjects, a total of 7274 were excluded: missing information on any MetS components at baseline survey (*n* = 4723); energy intake <800 or ≥4000 kcal/day in men and <500 or ≥3500 kcal/day in women (*n* = 4127); and missing BMI information (*n* = 78). Ultimately, 130,420 subjects including 43,682 men and 86,738 women remained in the final analysis (Figure 1). All study participants were given informed consent prior to entering the study. All participants voluntarily signed an informed consent form. As HEXA data are publicly open for use, the Institutional Review Board of the Seoul National University Hospital, Seoul, Korea approved it for statistical analysis (IRB No. E-1503-103-657).

### 2.2. Assessment of Egg Consumption

Diet was assessed using a 106-item self-administered Food Frequency Questionnaire (FFQ). The intake frequency of each food item had nine responses, ranging from “almost never” to “more than three per day” consumed during the past year. Three portion sizes were available: One-half standard serving, one standard serving, and one and a half servings. Subjects were asked on average, how often during the previous year they had consumed “whole eggs” (hence forward simply regarded as “eggs”) and how many eggs they had eaten on each occasion (units of consumption were 0.5, 1, and 1.5 eggs). We calculated the average daily amount of egg consumption per day by multiplying the frequency of consumption by specific portion size. Then average daily amount of egg consumption per day (one egg is 50 g) was converted to egg consumption frequency per week. The subjects were divided into five categories according to number of eggs consumed per week: <1, 1, 2–4, 5–6, and ≥7. Total energy and nutrient intake was calculated via a food composition table developed by the Korean Health and Industry of Development Institute [17]. The test for validity and reproducibility of our FFQ has been performed in a previous study [18]. Regarding validity, the deattenuated, age, sex, and energy intake-adjusted correlation coefficients between the FFQ and the 12-day diet records (DRs) ranged between 0.23 and 0.64 (median for all nutrients 0.39). Regarding reproducibility, the averages of correlations between the two FFQs 1 year apart were 0.45 for all nutrient intakes and 0.39 for nutrient densities.

### 2.3. Metabolic Syndrome Diagnosis

The current study defined MetS using the National Cholesterol Education Program Adult Treatment Panel III (NCEP-ATP III) [19], modified for the Asian guideline for waist circumference (WC). Participants who met three or more of the following criteria were classified as having metabolic syndrome: (1): waist circumference (WC) ≥90 and ≥80 cm for men and women, respectively; (2): triglycerides (TG) ≥150 mg/dL or drug treatment for elevated triglycerides; (3): high-density lipoprotein cholesterol (HDL-C) ≤40 and ≤50 mg/dL in men and women, respectively; (4): systolic blood pressure (BP) ≥130, diastolic BP ≥ 85 mmHg or drug treatment for elevated BP; and (5): fasting glucose ≥100 mg/dL or drug treatment for elevated fasting BP.

### 2.4. Covariates

Sociodemographic factors such as age, education, and menopausal status (women only) were included. Age was categorized into three groups: 40–49, 50–59, and 60–69 years old. Education was classified into three categories: middle school or below, high school graduate, and college or above. Menopausal status was classified as either post-menopausal women who have gone a year without menstrual flow or pre-menopausal women who currently experience monthly menstrual cycle. Smoking status was ascertained by posing the following question: “Have you smoked more than 20 packs of cigarettes (400 cigarettes) in your lifetime?” People who had smoked ≥400 cigarettes during their lifetime and still smoked cigarettes at the time of the survey were classified as current smoker; Subjects who responded as never having smoked 400 cigarettes were defined as non-smokers, and past-smoker were defined as those who had smoked ≥400 cigarettes during their lifetime but did not smoke at the time of the survey. Alcohol drinking history was determined by the following question: “Are you unable to consume alcohol or refuse to do so (for religious reasons, etc.)?” The respondents who have never drank alcohol were determined as never drinkers. On the other hand, drinkers who have answered the question “Do you still drink” with “Yes” were determined as current drinkers and “No” as former drinkers. Physical activity was assessed by posing the following question: “Do you do any sports regularly until you sweat?” Subjects who responded “Yes” to the question were assigned to the regular exercise group; the respondents who answered “No” were assigned to the non-regular exercise group.

### 2.5. Statistical Analysis

All analyses were performed separately by sex to investigate the sex-specific effect in the association between egg consumption and MetS. The chi-square test (for categorical variables) and linear regression (for continuous variables) were used to analyze the characteristics of the study population based on egg intake. A multivariable logistic regression model was used to assess whether egg consumption was independently associated with the odds for prevalent MetS by using the lowest category (<1 egg per week) as the reference. The 95% confidence intervals (95% CIs) of the odds ratios (ORs) were estimated using the Wald method. Multivariable stepwise analysis was conducted to determine a parsimonious model of logistic regression, with age, BMI, recruitment site, education, job, married status, smoking status, dinking status, regular exercise, energy intake, and menopausal status (only women). For both of men and women, age, recruitment site, education, smoking, alcohol drinking, regular exercise, and total energy intake were adjusted. We considered it necessary to adjust for BMI due to it being a strong risk factor for MetS and its components. As BMI is highly correlated with waist circumference, the final model excluded BMI from its WC OR calculation. The final multivariable model was adjusted for age (40–49, 50–59, and 60–69 years), BMI (continuous; except WC OR), recruitment site, education (≤elementary, middle, high, ≥college, and unknown), smoking (never, past, current, and unknown), alcohol drinking (non, current, and unknown), regular exercisers (yes, no, and unknown), and total energy intake (quartiles). Dietary factors (i.e., fruit, vegetables, and meat) showed high correlation with energy intake, and therefore were not included in the multivariable model. Additionally, adjustment for dietary factor (intake of fruit, vegetable, and meat) did not substantially alter the results. 

We assigned median values for each category and conducted a linear trend test. Subgroup analyses by age, BMI, smoking status (only men), alcohol drinking status, education, physical activity, and menopausal status (only women) were also performed. The significance of interaction terms was assessed by likelihood ratio tests with the use of a cross-product term. All *p*-values were two-sided, and statistical significance was set at below 0.05. All analyses were performed with SAS (version 9.4; SAS Institute Inc., Cary, NC, USA).

## 3. Results

The average egg intake of our participants was 12.0 g/day (12.2 g/day in men and 11.9 g/day in women). The characteristics of the 130,420 subject from the HEXA study based on egg consumption are presented in Table 1. In this cohort, 4.9% of men and 5.0% of women consumed ≥7 eggs/week and 45.7% of men and 48.0% of women consumed <1 egg/week. Compared with men who consumed <1 egg/week, those reporting ≥7 eggs/week were more likely to be current smokers and drinkers, and less likely physically active. Furthermore, it was found that women who consumed ≥7 eggs/week were younger, had lower BMI, were more likely to be current drinkers, and more physically active compared to those who consumed <1 egg/week. Both men and women who consumed ≥7 eggs/week had higher intakes of energy, carbohydrates, protein, fat, and cholesterol than those who consumed <1 egg/week, whereas energy from carbohydrates was inversely associated with egg consumption.

The prevalence of MetS was 26.1% (29.1% in men and 24.6% in women) among our participants. The association between egg consumption and the odds for MetS and its components are shown in Table 2. The prevalence of lower HDL-C (*p* < 0.0001), elevated blood pressure (*p* = 0.0112), and high fasting glucose (*p* = 0.0066) varied based on egg consumption in men. There were significant differences in the prevalence of five components of MetS based on egg consumption among women (all *p* < 0.0001). Using of <1 egg/week as the reference category, consumption of ≥7 eggs/week was associated with a statistically significant decreased odds for MetS in women (OR: 0.77, 95% CI: 0.70–0.84, *p* trend < 0.0001), but not in men (OR: 0.94, 95% CI: 0.84–1.05, *p* trend = 0.3806) when adjusted for all putative confounders.

Furthermore, consumption of ≥7 eggs/week in women was associated with decreased multivariable-adjusted ORs of elevated waist circumference (OR: 0.80, 95% CI: 0.75–0.86, *p* trend < 0.0001), hypertriglyceridemia (OR: 0.78, 95% CI: 0.72–0.85, *p* trend < 0.0001), low HDL-C (OR: 0.82, 95% CI: 0.77–0.88, *p* trend < 0.0001), elevated blood pressure (OR: 0.86, 95% CI: 0.80–0.92, *p* trend < 0.0001), and high fasting glucose (OR: 0.94, 95% CI: 0.83–0.99, *p* trend = 0.0015). In men, consumption of ≥7 eggs/week was associated with decreased multivariable-adjusted ORs for low HDL-C (OR: 0.89, 95% CI: 0.80–1.00, *p* trend = 0.0004). 

Adjusting for dietary factors such as intake of fruit, vegetables, and meat did not alter our results. (OR: 0.94, 95% CI: 0.84–1.06, *p* for trend = 0.4562 in men and OR: 0.77, 95% CI: 0.70–0.84, *p* for trend < 0.0001 in women). Moreover, excluding subjects with a history of type 2 diabetes, hypertension, and dyslipidemia; and excluding subjects who reported family history of type 2 diabetes and hypertension, did not affect the association of egg consumption with the odds for MetS.

In the subgroup analysis stratified by age group, BMI, smoking (only men), alcohol drinking, education, physical activity, and menopausal status (only women), egg consumption was found to be significantly associated with decreased ORs for MetS risk in men aged 60–69 years, with a BMI 18.0–25.0 kg/m^2^, and with education below college (Figure S1a). Among women, higher egg consumption was found to be significantly associated with the multivariable-adjusted decreased ORs for MetS in all subgroups strata (Figure S1b).

## 4. Discussion

In the current study, we identified that egg consumption was associated with lower odds of MetS prevalence among the Korean population. In particular, we have identified an inverse and linear relationship between egg consumption and the odds for MetS in Korean women. Regarding MetS components, egg consumption was associated with decreased odd ratios in all five components (elevated waist circumference, hypertriglyceridemia, reduced HDL-C, elevated blood pressure, and high fasting glucose) in women, and was associated with a decreased odd ratio of lower HDL-C level in men. 

Few studies have examined the association of egg consumption with the odds for MetS and its components. Although most previous studies regarding egg consumption have focused on type 2 diabetes or CVD as their outcome, the associations were inconclusive [7,20,21]. A previous meta-analysis, which included studies in North America, Asia, and Europe, found that an egg per day increased the risk of type 2 diabetes among the US population, whereas the association was not significant among Asian and European populations [22]. Furthermore, in a meta-analysis using 22 independent cohort studies, the association between higher egg consumption (≥1 egg/day) and the risk of CVD and cardiac mortality was not significant among non-diabetes patients [12]. In a Korean study of 2889 adults without chronic diseases, more than three eggs per week was significantly associated with a decreased risk of incidence for MetS in both men and women when compared to non-egg eaters [23]. 

The health benefits of egg consumption have been controversial. Some studies report the various essential nutrients (e.g., protein, fat-soluble and B vitamins, calcium, potassium, and choline) and bioactive components (e.g., lutein and zeaxanthin) with health benefits (i.e., improvements in lipoprotein metabolism and plasma carotenoid) [24]. Other studies have solely focused on cholesterol as the main component of eggs and reported increases in risk for CVD and type 2 diabetes [6]. Accordingly, many dietary guidelines restrict egg consumption on the basis that a medium-sized egg contain an average 200 mg of cholesterol, which is about 70% of cholesterol recommended for a healthy adult. Even though diet guidelines regarding egg consumption are commonly founded on their high cholesterol, previous studies on cholesterol consumption found that cholesterol from food had little impact on the lipid profile in the blood [25]. Moreover, cholesterol absorption efficiency from diet shows a large range from 15–85% between individuals [26]. Therefore, it is unlikely that egg consumption independently has an influence on blood lipid profiles.

Eggs, as a nutrient-dense food, contain high-quality protein, vitamins, minerals, and lower saturated fatty acids [27]. Additionally, eggs are rich in mono-unsaturated fatty acid (MUFA), poly-unsaturated fatty acid (PUFA), phospholipid, antioxidants (including lutein and zeaxanthin), and folate, which can improve insulin sensitivity and counteract disturbances in glucose metabolism [28,29]. Generally, as phospholipids effect blood lipid levels and primarily increase HDL-C, it is likely that eggs contribute to raising the plasma HDL-C levels [30]. In a previous intervention study, participants consuming whole eggs showed improvements in lipoprotein profiles, compared to those who consumed egg substitutes: increases in large LDL and HDL particles, greater reductions in total very-low-density lipoprotein (VLDL) and medium VLDL particles, and lecithin cholesterol acyltransferase (LCAT) activity [13]. Therefore, this may partially explain the inverse association between egg consumption and the odds of MetS in our study, as higher egg consumption was associated with a statistically significant decreased risk of reduced serum HDL-C in both men and women.

In the current study, we found no association between egg intake and the odds for MetS among men. First, gender difference may be due to the smaller sample size in men that may have limited the power to detect the association. Second, it is possible that the degree of completion of the FFQ was different between men and women [31]. Women may be more precise and reliable than men in completing the FFQ, as traditionally in Korea, women generally prepare the meals and are also more knowledgeable about diet and food. Third, the null finding in men may be in part attributable to residual confounding effects, particularly from smoking or alcohol drinking. In our study, the proportion of current smokers and drinkers was much higher in men than in women. Also, men who consumed more eggs (≥7 eggs/week) were more likely to be currently smoking, drinking, and less physically active, while women with high egg consumption were less likely to be currently smoking and more physically active. In previous studies that reported null [10,21] or positive [4] association between egg intake and risk of type 2 diabetes or CVD, subjects with higher egg consumption were more likely to smoke, drink, and be less physically active. On the other hand, other studies found an inverse association between egg consumption and type 2 diabetes[7] or MetS [23], wherein subjects with high egg consumption were less likely to smoke or more physically active. While our study adjusted for such lifestyle factors, it is possible that the residual confounding effects of smoking, alcohol drinking, and of other unmeasured factors on the risk of MetS resulted in the observed gender difference. Lastly, there may be possible genetic differences between men and women for diet-related pathology of MetS; specifically, the differences in sex chromosomes, sex-specific gene expression of autosomes, sex hormones, and their effects on organ systems [32].

There are several limitations to the current study. First, the results from this study cannot assess causality between egg consumption and MetS due to the cross-sectional design. However, in the present study, this association did not change after we excluded subjects who reported histories of type 2 diabetes, hypertension, and dyslipidemia. Nonetheless, prospective studies on egg consumption and subsequent risk of MetS are warranted to confirm finding from total subject of the present study. Second, the information on egg consumption was obtained via self-reported FFQ; thus, measurement errors in dietary assessment are inevitable and energy intake from FFQ may be less accurate compared with that from dietary record [33]. Additionally, we could not obtain information regarding cooking methods and mixed dishes containing eggs. Third, as in any observational study, although we carefully adjusted for the relevant confounders, we cannot entirely rule out the possibility of some unmeasured or residual confounding factors (e.g., dietary behavior, food preparation, and measurement error) associated with egg consumption as well as MetS. Despite these limitations, there are several strengths to the current study. Our study includes a considerably larger number of Korean adults and by using the HEXA-G database, it is a sample that has become more homogenous with stronger internal validity. We’ve also used a validated FFQ and standardized procedures to collect data, and adjusted for potentially crucial confounders to parse out the independent effect of egg consumption on MetS.

## 5. Conclusions

In conclusion, our results—based on a large-scale population study in Korea—demonstrate that higher egg intake was associated with lower odds of MetS and its components in women, and with lower odds of reduced HDL-cholesterol in men. The results of our study highlight the potential health benefits of egg consumption among adults in contrast to prior studies that have focused on the high cholesterol content of eggs. Additionally, we suggest that further research is needed among the general population and to confirm the results in a longitudinal study with a more precise diet-assessment method.

## Figures and Tables

**Table 1:** Baseline characteristics by egg consumption, the Health Examinees Study-Gem (HEXA-G) study, 2004–2013.

	Egg Consumption (Egg/Week)					*p*-Value *
	<1	1	2–4	5–6	≥7	
**Men (*n* = 43,682)**	19,939	13,798	6476	1334	2135	
Age (years) ^†^	54.5 ± 8.1	53.0 ± 8.4	52.1 ± 8.5	51.9 ± 8.4	54.0 ± 8.8	<0.0001
BMI (kg/m^2^)	24.4 ± 2.7	24.4 ± 2.8	24.5 ± 2.8	24.6 ± 2.8	24.4 ± 2.9	0.0290
College or above, *n* (%)	6469 (32.9)	5287 (38.8)	2671 (41.7)	596 (45.1)	964 (45.7)	<0.0001
Current smokers, *n* (%)	5783 (29.1)	4498 (32.7)	2277 (35.3)	478 (35.9)	672 (31.6)	<0.0001
Current drinkers, *n* (%)	14,143 (71.1)	10,192 (74.0)	4876 (75.4)	1008 (75.8)	1538 (72.4)	<0.0001
Regular exercisers, *n* (%)	11,296 (43.2)	7774 (43.6)	3761 (41.8)	758 (43.1)	1322 (37.9)	<0.0001
Dietary Intake ^‡^						
Energy (kcal)	1717.8 ± 435.6	1879.1 ± 445.6	2010.4 ± 479.2	2110.2 ± 515.4	2101.0 ± 506.8	<0.0001
Carbohydrate (g) ^‡^	312.1 ± 75.0	330 ± 76.8	342.5 ± 81.0	352.5 ± 86.5	346.6 ± 84.6	<0.0001
Protein (g) ^‡^	55.4 ± 20.1	63.9 ± 20.1	71.7 ± 22.2	77.2 ± 24.1	79.1 ± 25.3	<0.0001
Fat (g) ^‡^	24.9 ± 13.8	31.5 ± 14.1	37.1 ± 16.0	41.3 ± 17.3	42.4 ± 17.8	<0.0001
% Energy from carbohydrate	74.2 ± 6.9	71.4 ± 6.3	69.2 ± 6.6	67.8 ± 6.7	66.8 ± 7.0	<0.0001
% Energy from protein	12.9 ± 2.4	13.7 ± 2.2	14.3 ± 2.3	14.7 ± 2.3	15.1 ± 2.5	<0.0001
% Energy from fat	12.8 ± 5.0	15.0 ± 4.6	16.5 ± 4.8	17.5 ± 4.9	18.0 ± 5.0	<0.0001
Cholesterol (mg) ^‡^	108.5 ± 71.0	171.5 ± 70.1	250.3 ± 80.4	331.6 ± 84.1	420.9 ± 138.1	<0.0001
Fruit (g)	128.4 + 107.5	131.3 + 85.1	139.5 + 91.1	135.3 + 87.0	144.7 + 105.4	<0.0001
Vegetables (g)	142.7 + 147.6	148.0 + 126.1	146.8 + 128.4	145.0 + 123.5	158.7 + 136.6	<0.0001
Meat (g)	38.4 + 33.1	43.3 + 31.5	45.1 + 33.6	45.7 + 33.9	42.0 + 34.6	<0.0001
**Women (*n* = 86,738)**	41,639	25,771	12,409	2592	4327	
Age (years)	53.5 ± 7.6	51.6 ± 7.7	50.6 ± 7.8	50.3 ± 7.8	51.5 ± 7.8	<0.0001
BMI (kg/m^2)^	23.8 ± 2.9	23.5 ± 2.9	23.4 ± 3.0	23.5 ± 3.1	23.3 ± 3.0	<0.0001
College or above, *n* (%)	5922 (14.2)	5704 (22.1)	3246 (26.2)	707 (27.3)	1261 (29.1)	<0.0001
Current smokers, *n* (%)	896 (2.2)	518 (2)	285 (2.3)	79 (3.0)	93 (2.1)	0.0113
Current drinkers, *n* (%)	11,752 (28.2)	8383 (32.5)	4066 (32.8)	908 (35.0)	1328 (30.7)	<0.0001
Regular exercisers, *n* (%)	20,873 (50.1)	13,121 (50.9)	6520 (52.5)	1329 (51.3)	2432 (56.2)	<0.0001
Dietary Intake						
Energy (kcal)	1560.1 ± 457.3	1720.6 ± 462.2	1859.2 ± 497.9	1946.3 ± 531.5	1993.2 ± 541.8	<0.0001
Carbohydrate (g) ^‡^	287.5 ± 82.6	306.1 ± 82.5	320.9 ± 86.9	328.3 ± 90.8	331.5 ± 91.9	<0.0001
Protein (g) ^‡^	50.6 ± 20.0	58.7 ± 19.7	66.5 ± 21.8	71.8 ± 23.8	76.0 ± 25.8	<0.0001
Fat (g) ^‡^	21.3 ± 12.9	27.5 ± 13.3	33.1 ± 15.0	37.1 ± 16.3	39.6 ± 17.1	<0.0001
% Energy from carbohydrate	74.9 ± 7.2	71.9 ± 6.6	69.7 ± 6.7	68.0 ± 6.7	67.0 ± 6.9	<0.0001
% Energy from protein	13.0 ± 2.6	13.7 ± 2.3	14.4 ± 2.4	14.8 ± 2.4	15.3 ± 2.6	<0.0001
% Energy from fat	12.1 ± 5.1	14.3 ± 4.8	15.9 ± 4.9	17.1 ± 4.9	17.8 ± 4.9	<0.0001
Cholesterol (mg) ^‡^	101.5 ± 72.0	163.9 ± 68.8	244.2 ± 78.5	325.2 ± 84.0	415.7 ± 143.4	<0.0001
Fruit (g)	151.9 + 121.3	152.7 + 102.8	161.5 + 105.6	165.1 + 108.9	177.1 + 123.4	<0.0001
Vegetables (g)	203.9 + 191.4	208.0 + 167	207.4 + 162.7	200.4 + 162.0	221.3 + 173.0	<0.0001
Meat (g)	28.3 + 29.1	33.9 + 28.4	35.5 + 29.4	36.0 + 29.1	31.5 + 27.3	<0.0001

* *p*-Values for linear trend across egg consumption categories were calculated by chi-square tests for categorical variables and general linear regression for continuous variables. ^†^ Values are means ± SD or *n* (%). ^‡^ Nutrient intake values were energy adjusted using the residual method.

**Table 2:** Odd ratios (OR) * and 95% CI of metabolic syndrome and components according to egg consumption.

	Egg Consumption (Egg/Week)					*p*-Trend ^†^
	<1	1	2–4	5–6	≥7	
**Men (*n* = 43,682)**	19,939	13,798	6476	1334	2135	
MetS ^‡^	5825 (29.2) ^§^	3981 (28.9)	1912 (29.5)	388 (29.1)	595 (27.9)	
	Ref. (Reference)	0.97 (0.92–1.02)	1.00 (0.93–1.07)	0.96 (0.84–1.10)	0.94 (0.84–1.05)	0.3806
WC ≥90 cm	5698 (28.6)	4068 (29.5)	2010 (31.0)	377 (28.3)	615 (28.8)	
	Ref.	0.98 (0.93–1.03)	1.04 (0.97–1.11)	0.89 (0.79–1.01)	0.90 (0.82–1.00)	0.1327
Serum TG ≥150 mg/dL	7708 (38.7)	5446 (39.5)	2646 (40.9)	540 (40.5)	807 (37.8)	
	Ref.	0.98 (0.94–1.03)	1.01 (0.95–1.08)	0.97 (0.86–1.10)	0.94 (0.85–1.04)	0.4771
Serum HDL-C ≤40 mg/dL	4783 (24.0)	3135 (22.7)	1413 (21.8)	274 (20.5)	464 (21.7)	
	Ref.	0.94 (0.89–1.00)	0.90 (0.84–0.97)	0.82 (0.72–0.95)	0.89 (0.80–1.00)	0.0004
BP ≥130/85 mmHg	10,656 (53.4)	7171 (52.0)	3377 (52.2)	665 (49.9)	1118 (52.4)	
	Ref.	0.98 (0.93–1.02)	1.00 (0.94–1.06)	0.91 (0.81–1.02)	0.98 (0.89–1.07)	0.2943
Fasting glucose ≥100 mg/dL	7088 (35.6)	4679 (33.9)	2211 (34.1)	449 (33.7)	722 (33.8)	
	Ref.	0.97 (0.92–1.02)	1.00 (0.94–1.07)	0.97 (0.86–1.10)	0.99 (0.90–1.10)	0.7800
**Women (*n* = 86,738)**	41,639	25,771	12,409	2592	4327	
MetS	11,683 (28.1)	5836 (22.6)	2495 (20.1)	504 (19.4)	820 (19.0)	
	Ref.	0.90 (0.86–0.94)	0.83 (0.78–0.87)	0.80 (0.71–0.89)	0.77 (0.70–0.84)	<0.0001
WC ≥80 cm	18,627 (44.7)	10,137 (39.3)	4720 (38.0)	988 (38.1)	1533 (35.4)	
	Ref.	0.91 (0.87–0.94)	0.91 (0.87–0.95)	0.93 (0.85–1.01)	0.80 (0.75–0.86)	<0.0001
Serum TG ≥150 mg/dL	10,589 (25.4)	5540 (21.5)	2425 (19.5)	519 (20.0)	822 (19.0)	
	Ref.	0.90 (0.86–0.93)	0.83 (0.79–0.88)	0.86 (0.77–0.95)	0.78 (0.72–0.85)	<0.0001
Serum HDL-C ≤50 mg/dL	16,020 (38.5)	8895 (34.5)	3981 (32.1)	788 (30.4)	1332 (30.8)	
	Ref.	0.94 (0.91–0.97)	0.87 (0.83–0.91)	0.82 (0.75–0.89)	0.82 (0.77–0.88)	<0.0001
BP ≥130/85 mmHg	16,806 (40.4)	9197 (35.7)	4132 (33.3)	785 (30.3)	1368 (31.6)	
	Ref.	0.98 (0.94–1.01)	0.95 (0.91–0.99)	0.83 (0.76–0.91)	0.86 (0.80–0.92)	<0.0001
Fasting glucose ≥100 mg/dL	8868 (21.3)	4819 (18.7)	2121 (17.1)	425 (16.4)	739 (17.1)	
	Ref.	0.98 (0.94–1.02)	0.94 (0.89–0.99)	0.90 (0.81–1.01)	0.94 (0.83–0.99)	0.0015

* Adjusted for age (40–49, 50–59, and 60–69), BMI (continuous; not adjusted for WC OR), recruitment site, education (≤elementary school, middle school, high school, ≥college, and unknown), smoking (never, past, current, and unknown), alcohol drinking (non, current, and unknown), regular exercisers (yes, no, and unknown), and total energy intake (quartiles). ^†^ Linear trends across categories of egg consumption were tested using the median consumption value for each categories as an ordinal variable. ^‡^ MetS: The presence of three or more of the following components: (1): waist circumference (WC) ≥90 cm in men and ≥80 cm in women; (2): high triglyceride level (TG) ≥150 mg/dL; (3): low high density lipoprotein cholesterol (HDL-C) level <40 mg/dL in men and <50 mg/dL in women or taking anticholesterol medication; (4): high blood pressure (BP) ≥130/85 mmHg or taking antihypertensive medicine; (5): high fasting glucose level ≥100 mg/dL or taking medication to treat diabetes mellitus. ^§^ The number of cases (percentage).

## Supplementary Materials

The following are available online at www.mdpi.com/2072-6643/9/7/687/s1, Figure S1: Subgroup analyses for the association between egg consumption and prevalence of MetS in men (**a**) and women (**b**), stratified by age, BMI, alcohol drinking, education, physical activity, and menopausal status. Model was adjusted for age (40–49, 50–59, and 60–69), recruiting site, education (≤elementary school, middle school, high school, ≥college, and unknown), smoking (never, past, current, and unknown), alcohol drinking (non, current, and unknown), physical activity (yes, no, and unknown), and total energy intake (quartiles). The ORs (95% CIs) of subjects with high egg consumption (≥ 7 eggs/week) was calculated with those consuming <1 egg/week.
