# Supplementary material for: Egg Consumption and Risk of Metabolic Syndrome in Korean Adults: Results from the Health Examinees Study
Source: Nutrients. 2017 Jul 2;9(7):687. doi: 10.3390/nu9070687 (PMC5537802; doi:10.3390/nu9070687)
Supplement: Supplementary file 1 [file nutrients-09-00687-s001.pdf]

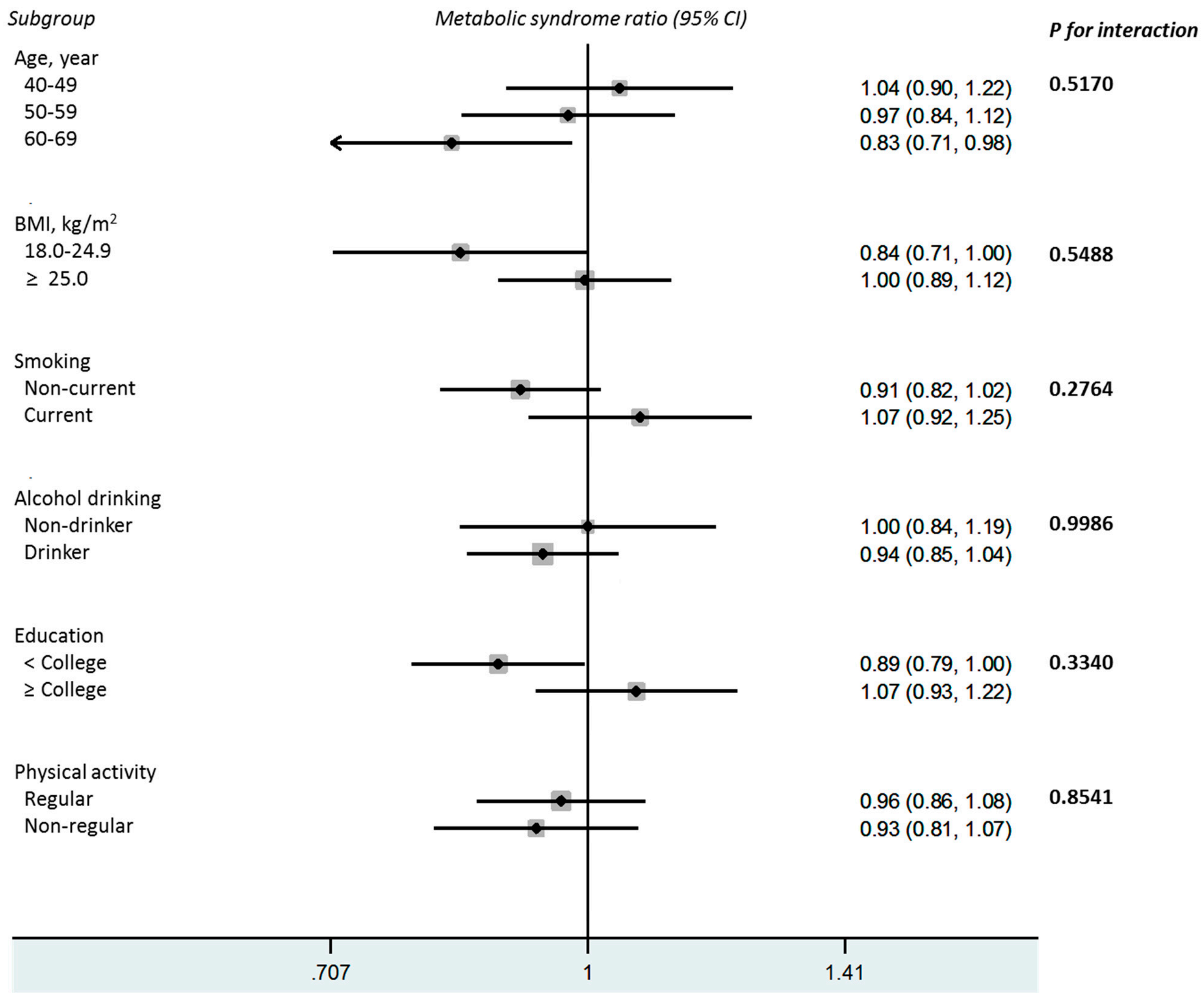

Supplement Figure 1a. Subgroup analyses for the association between egg consumption and prevalence of MetS in men, stratified by age, BMI, smoking, alcohol drinking, education, and physical activity. Model was adjusted for age (40–49, 50–59, and 60–69), recruitment site, education (≤elementary school, middle school, high school, ≥college, and unknown), smoking (never, past, current, and unknown), alcohol drinking (non, current, and unknown), physical activity (yes, no, and unknown), and total energy intake (quartiles). The ORs (95%CIs) of subjects with high egg consumption (≥ 7 eggs / week) was calculated with those consuming <1 egg/week.

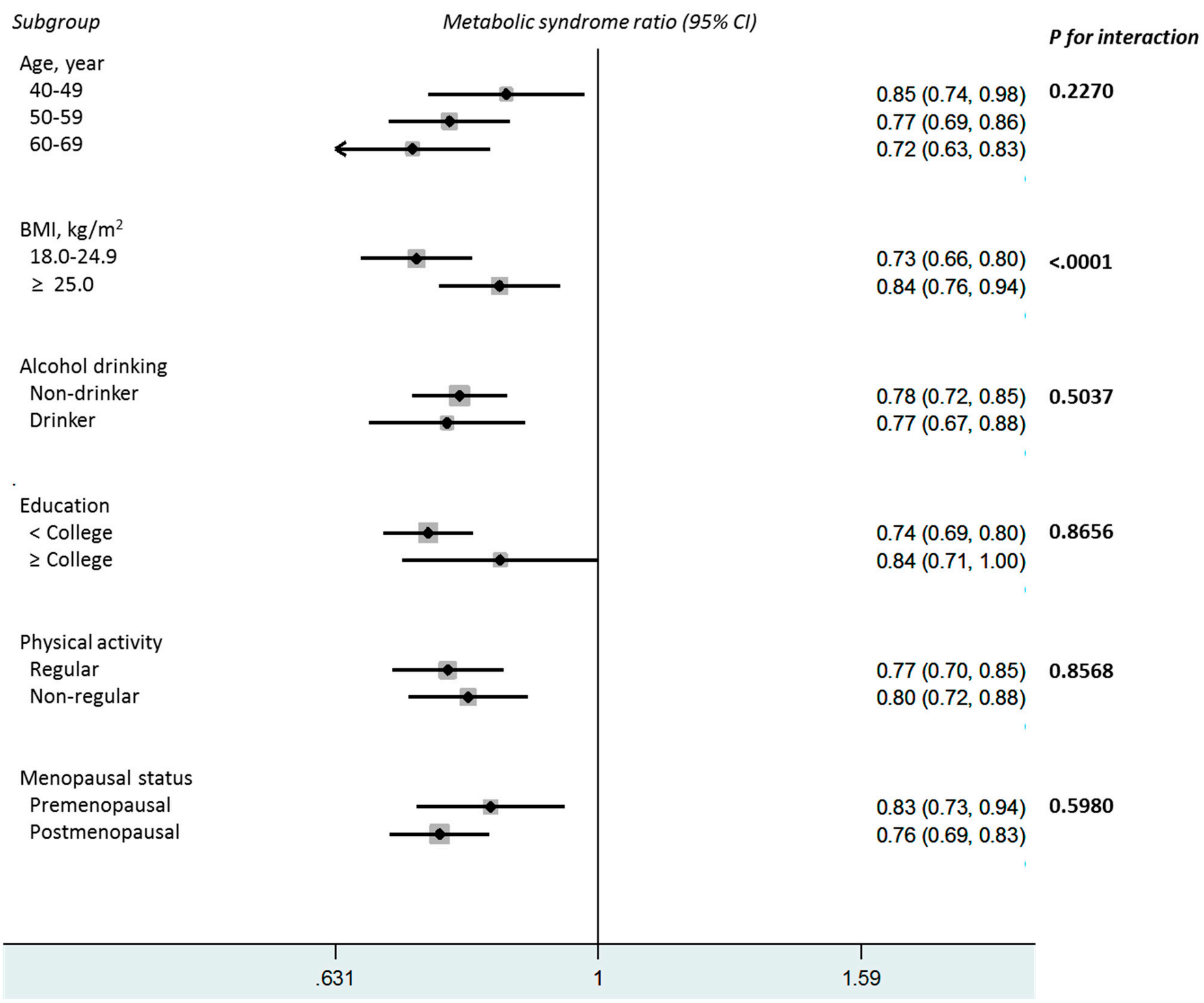

Supplement Figure 1b. Subgroup analyses for the association between egg consumption and prevalence of MetS in women, stratified by age, BMI, alcohol drinking, education, physical activity, and menopausal status. Model was adjusted for age (40–49, 50–59, and 60–69), recruiting site, education (≤elementary school, middle school, high school, ≥college, and unknown), smoking (never, past, current, and unknown), alcohol drinking (non, current, and unknown), physical activity (yes, no, and unknown), and total energy intake (quartiles). The ORs (95%CIs) of subjects with high egg consumption (≥ 7 eggs / week) was calculated with those consuming <1 egg/week.
